# An interactive data visualisation application to investigate nosocomial transmission of infections

**DOI:** 10.12688/wellcomeopenres.15240.2

**Published:** 2019-08-20

**Authors:** Catherine M. Smith, David J. Allen, Sameena Nawaz, Zisis Kozlakidis, Eleni Nastouli, Andrew Hayward, Katherine N. Ward

**Affiliations:** 1 Institute of Health Informatics, University College London, London, NW1 2DA, UK; 2 Department of Infection Biology, Faculty of Infectious and Tropical Diseases, London School of Hygiene & Tropical Medicine, London, WC1E 7HT, UK; 3 Virus Reference Department, Public Health England, London, NW9 5HT, UK; 4 NIHR Health Protection Unit in Gastrointestinal Infections, London, UK; 5 World Health Organization, International Agency for Research on Cancer, Lyon, France; 6 Clinical Virology, University College London Hospitals NHS Foundation Trust, London, NW1 2BU, UK; 7 Department of Population, Policy and Practice, UCL GOS Institute of Child Health, University College London, London, UK; 8 Institute of Epidemiology and Health Care, University College London, London, WC1E 6BT, UK; 9 Division of Infection and Immunity, University College London, London, WC1E 6BT, UK

**Keywords:** Norovirus, infection control, cross infection, software, outbreak, virus genomics

## Abstract

**Background: **Healthcare-associated infections represent a major threat to patient, staff and visitor safety. Identification of episodes that are likely to have resulted from nosocomial transmission has important implications for infection control. Routinely collected data on ward admissions and sample dates, combined with pathogen genomic information could provide useful insights. We describe a novel, open-source, application for visualising these data, and demonstrate its utility for investigating nosocomial transmission using a case study of a large outbreak of norovirus infection.

**Methods: **We developed the application using Shiny, a web application framework for R. For the norovirus case study, cases were defined as patients who had a faecal sample collected at the hospital in a winter season that tested positive for norovirus. Patient demographics and ward admission dates were extracted from hospital systems. Detected norovirus strains were genotyped and further characterised through sequencing of the hypervariable P2 domain. The most commonly detected sub-strain was visualised using the interactive application.

**Results: **There were 156 norovirus-positive specimens collected from 107 patients. The most commonly detected sub-strain affected 30 patients in five wards. We used the interactive application to produce three visualisations: a bar chart, a timeline, and a schematic ward plan highlighting plausible transmission links. Visualisations showed credible links between cases on the elderly care ward.

**Conclusions: **Use of the interactive application provided insights into transmission in this large nosocomial outbreak of norovirus, highlighting where infection control practices worked well or could be improved. This is a flexible tool that could be used for investigation of any infection in any hospital by interactively changing parameters. Challenges include integration with hospital systems for extracting data. Prospective use of this application could inform better infection control in real time.

## Introduction

Healthcare-associated infections (HCAI) represent a major threat to patient, staff and visitor safety. The average prevalence of HCAI has been estimated at 4–6% in the USA and Europe, and impacts include prolonged hospital stays, additional financial costs, and excess deaths ^1– 3^. The term HCAI may refer to infections caused by a range of pathogens either contracted within the healthcare setting or brought in from the community. Identifying episodes that are likely to have resulted from nosocomial transmission has important implications for infection control.

Infections are often classified as ‘hospital-acquired’ if they were identified after a certain number of days have passed following admission to hospital. The number of days required to meet this threshold varies depending on the incubation period of each pathogen. For example, Public Health England classifies *Clostridium difficile* infection as hospital-acquired if the specimen is taken on or after the fourth day of admission, and *Staphylococcus aureus* bacteraemia if the specimen is taken on or after the third day of admission ^4^. There is no formal consensus for classification of norovirus infection, which may therefore vary according to local infection control protocols. Although these classifications are useful for identification of broad trends and surveillance, more detailed information is required to make specific inferences about nosocomial transmission events or to track outbreaks.

Evidence for potential nosocomial transmission may be derived from epidemiological and pathogen genomic data. For example, the dates on which patients were admitted to different wards in the hospital can be used to find temporal and spatial overlaps between cases. Combining this with knowledge of the natural history of the infection (such as the incubation and infectious periods of the aetiological agent) can then determine whether these overlaps represent plausible windows in which transmission may have occurred. Incorporating genomic information about the pathogens causing infections and associated with the outbreak can indicate whether they are likely to be part of the same outbreak, or if separate introductions are more probable. These types of data are increasingly becoming routinely available, but combining them into a form that can be interrogated easily and used as part of an infection control response is challenging.

Visualisation of data can provide an easy to interpret means of assessing complex data sets ^5, 6^. Interactive visualisations, in particular, allow data to be explored without requiring pre-defined hypotheses. This would be valuable in the context of HCAIs to synthesise information on type, place and time of infection and aid investigation of outbreaks. Here, we describe a novel, open-source, visualisation application, HospMapper ^7^, and demonstrate its utility for investigating nosocomial transmission using a case study of a large outbreak of norovirus infection.

### Case study: Norovirus outbreak investigation

Human noroviruses are a group of genetically diverse pathogens associated with gastrointestinal disease that is generally characterised by sudden onset nausea and vomiting, and may be accompanied by other symptoms including watery diarrhoea and fever ^8^. Outbreaks of norovirus-associated gastroenteritis have the most impact in healthcare settings, where they can become protracted, often affecting multiple wards and both patients and staff. In England and Wales, such outbreaks have been estimated to result in >£100 million in conventional costs for the NHS annually, and an estimated total economic burden of >£295 million ^9^.

This is a case study of a large norovirus outbreak that occurred in a single hospital during a winter season. We aimed to describe the molecular and epidemiological characteristics of the outbreak, and to visualise it using the HospMapper application ^7^.

## Methods

### Application design

The application is designed to display data about hospital in-patients who have tested positive for an infection of interest. The minimum (‘core’) data set required to run the application comprises the patient admission date, the date that their positive sample was taken, and the ward they were on when the sample was taken. The core data set can be supplemented with additional descriptive characteristics, such as patient age, sex, or the identifier of a genetically defined cluster. Optional data sets, which enable additional visualisations, are ward transfers (dates that patients move into and out of each ward during their hospital admission) and genetic distances (pairwise genetic distances between pathogens infecting each two patients).

The application can produce three interactive visualisations in different tabs: a bar chart (‘epidemic curve’), a timeline, and a schematic ward plan. Data in each tab can be filtered and colour coded according to ward, sample date, and patient characteristics. Infections can be classified as hospital- or community-acquired based on the sample and admission dates. The number of days of admission prior to the sample required for an infection to be classified as hospital-acquired is defined by a user-controlled slider.

In the timeline and ward plan tabs, which both show patients’ locations throughout their admission, cases can also be colour coded according to their likely period of infection over time. This ‘infection period’ is classified as either pre-exposure to the infection; during the likely exposure period; the likely incubation period; the likely infectious period; or post-infectious period. It is defined based on the date of the positive sample and estimates of the incubation period, sampling delay, and infectious period of the specific infection being investigated, which are specified by user inputs. A diagram showing how the components of the infectious period are calculated from the inputs is shown in the ‘About’ tab ( *Extended data*: Additional file 1) ^10^.

In the ward plan visualisation, there is a ‘Links’ tab, with options to show plausible epidemiological links between patients based on the infection period. ‘Epidemiological link (ever)’ draws links between patients who, at any time during their admissions, are in the same ward when one patient is in their infectious period and the other in their exposure period. Selecting a patient who is in their exposure period and checking ‘Potentially infected by’ will draw links to all other patients who are in their infectious period and in the same ward on that day. Selecting a patient who is in their infectious period and checking ‘Potentially infected’ will show links to all other patients who are in their exposure period and in the same ward on that day. There is also an option to include, in the ward transfers file, a variable that represents which floor of the hospital each ward is located on. Based on this number, the application will display the wards arranged by floor in the plan visualisation. Bed locations within a ward could also be represented using these parameters, if this information is available. This allows the user to set up the schematic to more closely resemble the layout of the hospital. 

### Implementation

We developed the interactive application using Shiny v1.2.0, a web application framework for the statistical software, R ^11, 12^.

### Operation

The code for the application is open-source and available on GitHub, which also includes a user guide. To run the application from a local machine, R needs to be installed with the Shiny package loaded. A single command can then be used to download the code for the application from GitHub and launch it in an interactive browser window. A demonstration version of the app is available at https://cathsmith57.shinyapps.io/HospMap/, which includes ‘dummy’ data that can be used to test the app. *Extended data*: Additional file 2 ^13^ demonstrates the data import process: After selecting the file, a preview is displayed. The user must select the name of the column in the data set that represents each variable required to run the application. Pressing ‘Run’ then produces the interactive visualisations.

### Case study: Norovirus outbreak investigation

***Hospital setting and case definition*.** The outbreak occurred during winter months in a tertiary hospital with 665 in-patient beds which provides accident & emergency, cancer care, critical care, general medicine and paediatric care amongst other services. The wards are organised into bays containing 4–6 beds, and all have some single-bedded side-room capacity. Cases were defined as patients who had a faecal sample collected at the hospital in the winter season that tested positive for norovirus by real-time reverse-transcription PCR (RT-qPCR). Patient demographics (age and sex), dates of admission, sample dates, and transfers between wards were extracted from hospital systems ^14^. We have altered the dates displayed in figures in this paper to preserve patient anonymity.

Ethical approval was not required for this study because it was undertaken as part of outbreak investigation and management, and personal identifiable information data were not used.

***Molecular characterisation*.** Samples were tested during the outbreak at the hospital’s clinical virology laboratory by RT-qPCR directed at the ORF1/2 conserved region ^15^. A subset of specimens were then retrospectively analysed at the Virus Reference Department, Public Health England to confirm that norovirus RNA was still detectable, before further characterisation as described below.

Detected norovirus strains were genotyped as previously described ^16^, through analysis of nucleotide sequence representing the S-domain of the VP1 protein. For GII.4 norovirus strains, further characterisation was performed through sequencing of the hypervariable P2 domain of the VP1 protein, as previously described ^17^. This method has been shown to provide sufficient resolution to identify intra-genotypic variants, and to discriminate between strain transmission within an outbreak and separate introduction or events ^18^. Sequence analysis, generation of consensus sequences and pairwise alignments of sequences were performed using Bionumerics v6.1 (Applied Maths, Kortijk, Belgium). MEGA software is an open access alternative that could also be used for this analysis.

***Epidemiological analysis*.** We described the age and sex distribution of cases. We classified cases as hospital-acquired if their first positive specimen was taken on or after the third day of admission and calculated the proportion of cases that met this definition. We plotted the cases according to genotype (and sub-strain for GII.4 isolates) and sampling ward on a timeline. For the most commonly detected sub-strain of GII.4, we extracted dates that patients moved between wards from hospital systems for further visualisation in the HospMapper application ^7^.

***Visualisation using HospMapper application*.** We imported data from the most commonly detected sub-strain (GII.4-D) of norovirus cases into the HospMapper application ^7^. This involved loading the ‘core’ and ‘ward transitions’ data sets, and specifying the relevant variables in the data input step. We used the application to visualise the occurrence of norovirus cases in this sub-strain over time, in different wards, and to identify potential transmission links.

## Results

### Case study: Norovirus outbreak investigation

**Figure 1.**
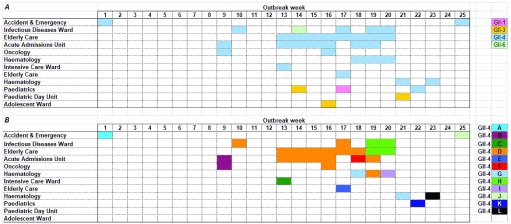
Timeline of 25-week outbreak period by ward and norovirus strain type. ( **A**) All strains. Colours represent norovirus strain types determined by standard strain typing. ( **B**) GII.4 strain only. Colours represent norovirus sub-strain types determined by analysis of the hypervariable P2 domain.

Across the six month period, 155 norovirus-positive specimens were collected from 107 patients. The median patient age was 71.7 years (range: 0.4–99.3 years), and the male-to-female ratio among patients was similar (51 (48%) male, 56 (52%) female). These specimens represented 111 episodes of illness among the 107 sampled patients ( Figure 1). The majority (67 episodes, 60%) of these 111 episodes were classified as hospital-acquired (first positive specimen taken on or after the third day of admission).

Of the 155 norovirus-positive specimens, a subset of specimens (n=86) from 67 patients were available for further characterisation in a retrospective analysis, from which norovirus RNA could still be detected in 70 specimens (81%) from a total of 51 patients. The remaining 16 samples (from 16 patients) from which norovirus RNA was no longer detectable were excluded from further analysis.

Amongst the 51 patients in whom the presence of norovirus RNA was confirmed, the majority (46/51, 90.2%) were typed as GII.4, with the remaining being GII.3 (3/51, 5.9%), GII.1 (1/51, 2.0%) and GII.6 (1/51, 2.0%) ( Figure 1A). Further characterisation of the GII.4 strains by sequence analysis of the hypervariable P2 domain revealed a total of 12 GII.4 sub-strains, designated A to L, on the basis of 100% nucleotide identity within the P2 hypervariable region ( Figure 1B). There were, therefore, at least 15 genetically distinct norovirus sub-strains in the hospital in this study period (4 strains, one of which included 12 sub-strains). The most commonly detected sub-strain (GII.4D) affected 30 patients (65.2% of the 46 GII.4 strains).

### Visualisation using HospMapper application

**Figure 2.**
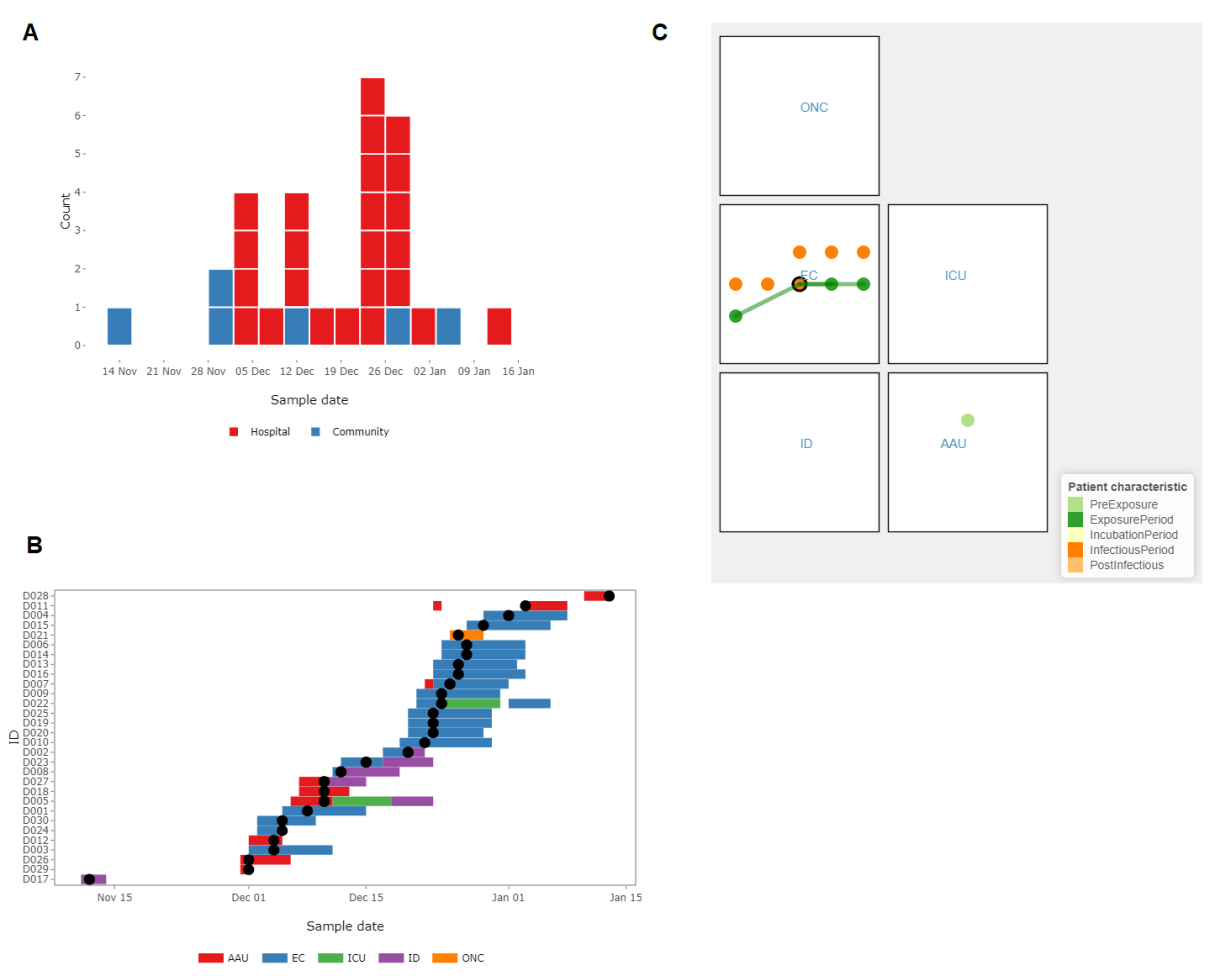
Example visualisations from HospMapper application of GII4.D norovirus cluster. ( **A**) Epidemic curve. Each box represents a patient. Colours show hospital- or community-acquired infections (hospital-acquired if first positive specimen taken on or after the third day of admission). ( **B**) Timeline. Colours represent ward, dots represent dates of first positive sample. ( **C**) Schematic plan of hospital wards. Boxes represent hospital wards. Dots represent patients who were staying on the ward a selected date. Colours represent period of infection, based on sample date and assuming an incubation period of 1–4 days, a sampling delay of 1 day and infectious period of 4 days. Lines show plausible epidemiological links between circled case and others in the ward on the same day. NB: Dates have been altered to preserve anonymity.

We imported data from the 30 patients with the GII.4D strain into the HospMapper application ^7^. In the resulting interactive bar chart, sample date is shown on the x axis and each box on the chart represents one person ( *Extended data*: Additional file 3 ^19^; Figure 2a). We used the ‘Colour by patient characteristics’ option to shade the boxes according to whether the infection was classified as community or hospital-acquired, and defined a hospital-acquired infection as a sample taken at least two days after admission using the slider. This showed that the first three cases in the cluster were classified as community-acquired, and the majority thereafter were hospital-acquired.

After removing the community-acquired infections from the chart, we changed the options so that the boxes were coloured according to the ward on which the patient’s positive sample was taken. This showed that the hospital-acquired infections were mostly diagnosed on the EC (elderly care) ward, with the remainder on the AAU (acute admissions unit). Adding community-acquired cases back on to the chart showed that additional community-acquired cases were diagnosed on the ID (infectious diseases) and oncology wards.

The interactive timeline visualisation displays, in each row, a patient’s duration of stay in the hospital ( *Extended data*: Additional file 4 ^20^; Figure 2b). We used the options to order the patients in the timeline according to the date on which their positive sample was taken, and to colour code the timeline according to the ward that the patient was in on different days during their admission. This highlighted two clear groups of cases sampled around the same time on AAU and EC wards. It also showed that a number of cases moved into the ID ward shortly after their positive sample was taken.

We then filtered the data so that only patients who spent time on the EC or AAU wards were displayed, and changed the timeline so that it was coloured by the ‘infection period’ of each case. We set the incubation period as ranging from one to three days, the sampling delay as one day, and infectious period as seven days. This showed that exposure (dark green) periods of several patients in EC overlapped in time with infectious (dark orange) periods of other patients on the same ward. By contrast, there was no overlap in the exposure and infectious periods amongst the group of patients on the AAU.

The schematic plan visualisation of the hospital wards shows dots representing each of the patients who were in the ward on a given date ( *Extended data*: Additional file 5 ^21^; Figure 2c). We focused first on the AAU ward. As suggested in the timeline, all patients in this ward were in the same infection period at the same time, and there were therefore no suspected epidemiological transmission links between them. Later in the outbreak, there were many potential epidemiological links between cases in the EC ward.

## Discussion

We have developed a novel application, HospMapper ^7^, which produces visual displays to aid interpretation of complex epidemiological and genomic data in hospital settings. It can be used to identify areas in the hospital in which infections may have been transmitted, and to trace possible transmission links by highlighting patients that share epidemiological and/or pathogen genetic links. The application may be used by infection control teams or researchers to improve understanding of transmission of infections in hospitals and guide infection control measures.

As a case study, we have described the molecular and epidemiological characteristics of an outbreak of norovirus across a winter season in a hospital. Through standard norovirus genotyping and P2 domain strain typing, we found that this large outbreak was made up of isolated incidents caused by at least 15 unrelated viruses. There was evidence of protracted onward transmission for one sub-strain (GII.4D), lasting 16 weeks and affecting at least 30 patients. There are various factors that may have contributed to this strain persisting in the hospital whilst others did not. These include factors relating to the virus strain and its ability to survive in the environment, the patient groups affected, and the architecture and staffing of the hospital.

We demonstrated the use of HospMapper to investigate the most commonly detected sub-strain. These visualisations have contributed to understanding of how the infection is likely to have been transmitted through the hospital, with implications for infection control and public health: Within the most commonly detected sub-strain, many of the cases were identified on the same ward (elderly care). Whilst these cases were not all present on the ward at the same time, there are credible epidemiological links which indicates that it is plausible that they were part of the same transmission chain. Cases were also transferred through the infectious diseases and intensive care units, but the visualisations were not suggestive of onward transmission on these wards. There were no clear epidemiological transmission links between cases identified on the acute admissions unit, although cases of the same strain continued to be detected over time. Transmission on this ward may therefore have occurred through unidentified cases (for example, individuals who did not stay in the hospital long enough to present as a case, patients with undiagnosed infection, or asymptomatic staff or visitors who were not sampled), or an environmental reservoir ^18^. These observations indicate that infection control procedures on highly specialised wards (infectious disease and intensive care) were robust, but that more generalised wards or those with patients transferring in and out regularly (elderly care and acute admissions unit) continue to pose a challenge for outbreak management.

Although this study provided interesting data on the dynamics of norovirus in this hospital, it is limited by being retrospective: It is likely that not all patients affected during the outbreak were sampled, particularly after a norovirus outbreak had been declared. Only a subset of samples were available for further characterisation, and we were unable to detect norovirus RNA in all samples at re-analysis. This was most likely to be related to low viral loads and/or template degradation in stored nucleic acid extracts used where original specimens were no longer available. The availability of clinical samples that accurately represent all cases and epidemiological data accurately describing patient and staff movements during the outbreak may not be possible to collect in all incidences. Additionally, information on differences in severity of disease, duration of virus shedding, virus load and symptoms of disease may not always be collected and can be difficult to obtain retrospectively. However, together these factors are likely to have an important role in transmission during an outbreak.

The main advantages of using the HospMapper software are its interactivity and flexibility. Although in this study we have used it to investigate an outbreak of norovirus, it could be used for any infection, with the capability to adjust parameters to appropriate values. It can also be used for any hospital or healthcare setting, as the depiction of wards is calculated automatically by the software. The interactivity allows data to be explored quickly and without *a priori* hypotheses. It also means that the impact of assumptions about the data can be tested easily, for example, by adjusting the number of days of admission required for infections to be classified as hospital-acquired.

A further advantage is the use of the open source R software and Shiny application framework. Knowledge of R is not required to run the application, but the code is free to download and could be customised or extended by users familiar with the software. The application also does not require complex computational infrastructure, and could therefore be used even in remote or decentralised healthcare settings. Shiny applications are run locally and do not require upload of any patient information to the internet, thereby ensuring that there are no issues with security of potentially-sensitive information. Alternatively, users may choose to upload a version of the application on a local or remote server, and use appropriate firewalls or authentication services to protect their data. This would enable the application to be used without the need to install R.

There are also limitations to this software. Primarily, as a free application, it is not supported, and troubleshooting will therefore have to be carried out by users. We have, however, provided a user guide and dummy data sets to help with setting up the application. Another challenge is integration with existing hospital systems to facilitate data import, as some expertise will be required to extract data that is appropriately formatted. Finally, as with any data visualisations, it must be noted that insights provided by the application represent hypotheses, which will still require formal testing with classical epidemiological and/or microbiological methods.

In future, we envisage that this software could be used prospectively by infection control teams. Using regularly updated data sets, for example over the course of a winter season, could allow infections to be tracked through hospitals in real-time, enabling improved targeting of infection control measures, and lessening the impact of outbreaks. For example, the capability offered by the software to map locations of wards/beds of patients and analyse their movements creates the opportunity to assess the impact of spatial proximity during an outbreak. This has been shown to be important for understanding transmission in norovirus outbreaks ^22^. Although this feature was not used for the norovirus case study, the software can be used to explore genome sequencing data, which will be of increasing importance when high-throughput whole genome sequencing is used routinely. This tool could also provide a way to evaluate interventions for the control and prevention of infections such as norovirus in hospital setting and provide evidence to inform future guidelines.

## Data availability

### Underlying data

Figshare: Demonstration data. https://doi.org/10.6084/m9.figshare.8241530.v2 ^14^.

This project contains the following underlying data:

- Noro_core_demo.csv (core demonstration data for investigation of norovirus outbreak using the interactive timeline visualisation in the HospMapper application).
- Noro_wardtrans_demo.csv (dates that patients move into and out of each ward).

### Extended data

Figshare: Additional file 1. https://doi.org/10.6084/m9.figshare.7970921.v1 ^10^. Screencast movie demonstrating calculation of the ’infection period’ based on user inputs in the ‘About’ tab of HospMapper application.

Figshare: Additional file 2: https://doi.org/10.6084/m9.figshare.8063288.v1 ^13^. Screencast movie demonstrating process for importing data into HospMapper application.

Figshare: Additional file 3. https://doi.org/10.6084/m9.figshare.8063300.v1 ^19^. Screencast movie of investigation of norovirus outbreak using the interactive bar chart visualisation in the HospMapper application. AAU, acute admissions unit; EC, elderly care; ICU, intensive care unit; ID, infectious diseases; ONC, oncology.

Figshare: Additional file 4. https://doi.org/10.6084/m9.figshare.8063303.v1 ^20^. Screencast movie of investigation of norovirus outbreak using the interactive timeline visualisation in the HospMapper application. AAU, acute admissions unit; EC, elderly care; ICU, intensive care unit; ID, infectious diseases; ONC, oncology.

Figshare: Additional file 5. https://doi.org/10.6084/m9.figshare.8063309.v1 ^21^. Screencast movie of investigation of norovirus outbreak using the schematic ward plan visualisation in the HospMapper application. AAU, acute admissions unit; EC, elderly care; ICU, intensive care unit; ID, infectious diseases; ONC, oncology.

Please note that to ensure anonymity, dates in the underlying and extended data have been modified.

Data are available under the terms of the Creative Commons Attribution 4.0 International license (CC-BY 4.0).

## Software availability

**Source code available at:** https://github.com/cathsmith57/HospMap

**App demonstration version at:** https://cathsmith57.shinyapps.io/HospMap/

**Archived source code at time of publication:** https://doi.org/10.5281/zenodo.2634073 ^7^.

**License:** GNU General Public License, version 3.

## Author information

C.M.S. and D.J.A. are joint first authors; A.H. and K.N.W. jointly supervised this work.
