# Supplementary material for: An interactive data visualisation application to investigate nosocomial transmission of infections
Source: Wellcome Open Res. 2019 Aug 20;4:100. Originally published 2019 Jun 24. [Version 2] doi: 10.12688/wellcomeopenres.15240.2 (PMC6668043; doi:10.12688/wellcomeopenres.15240.2)
Supplement: Peer review history

# Peer review: Reviewer response for version 1

**DOI:** 10.21956/wellcomeopenres.16634.r35982

**Published:** 2019-07-30

**Authors:** Ben Lopman

**Affiliations:** 1 Department of Epidemiology, Rollins School of Public Health, Emory University, Atlanta, GA, USA

In this report, Smith and coauthors clearly describe a neat visualization tool, which they call HospMapper. The tool is an R Shiny app, so is free and open source. The software is available on GitHub along with documentation and the example data  are on FigShare. The authors make a good case that this will be a useful tool for infection control teams in that they will be able to visualize ongoing or historic outbreaks and in doing so, better understand transmission patterns and/or generate testable hypotheses. The app can handle and display a range of patient level data including crude spatial information (ward location) as well as genomic data. These characteristics allow for graphing the time series of the outbreak and can illustrate spatial patterns. A worked example of a large outbreak (or series of outbreaks) of norovirus in a UK hospital. The app is clearly described and a strong case is made that it can be applied to a range of HCAIs.

 However, I would like to have seen more work, or at least suggestions, regarding how this software could be integrated into 'classical' or spatial statistical analysis of outbreaks. An additional nice feature would be the ability to import a hospital/ward map and the cases be plotted on the map using coordinated. Is that possible or something that may be added in the future?

I confirm that I have read this submission and believe that I have an appropriate level of expertise to confirm that it is of an acceptable scientific standard.

# Peer review: Reviewer response for version 1

**DOI:** 10.21956/wellcomeopenres.16634.r35980

**Published:** 2019-07-24

**Authors:** Sofie Elisabeth Midgeley

**Affiliations:** 1 Virus Surveillance and Research Section, Statens Serum Institut, Copenhagen, Denmark

The article describes the development and testing of a novel software tool for the visualisation and investigation of transmission of infectious agents in a hospital setting. The authors clearly describe the rationale for development of the tool; to make the process of investigating nosocomial outbreaks easier. All the included parameters are clearly described, as well as instructions on how to define and adapt them to different settings. The tool has been developed as open-source, and clear instructions on how to access it and operate it are included. Data interpretation is demonstrated using a case study. The outputs are clear and intuitive, and easy to read. The case study also shows how the parameters can be adapted to different hospitals and wards. The case study clearly demonstrates how potential transmission chains can be identified and investigated.

 The authors also clearly describe the limitations of their new tool.

I confirm that I have read this submission and believe that I have an appropriate level of expertise to confirm that it is of an acceptable scientific standard.
